# 4D-printed intelligent reflecting surface with improved beam resolution via both phase modulation and space modulation

**DOI:** 10.1038/s41378-024-00795-1

**Published:** 2024-10-29

**Authors:** Kyounghwan Kim, Ratanak Phon, Eiyong Park, Sungjoon Lim

**Affiliations:** 1 Department of Intelligent Semiconductor Engineering, Chung-Ang University, Seoul, 06974 Republic of Korea; 2 Department of Electrical and Computer Engineering, Stevens Institute of Technology, Hoboken, NJ 07030 USA; 3 School of Electrical and Electronic Engineering, Chung-Ang University, Seoul, 06974 Republic of Korea

**Keywords:** Electrical and electronic engineering, Nanophotonics and plasmonics

## Abstract

Recently, intelligent reflecting surfaces (IRSs) have emerged as potential candidates for overcoming the line-of-sight issue in 5 G/6 G wireless communication. These IRSs can manipulate the direction of reflected beams, enabling efficient beam steering to enhance the performance of wireless communication. Each unit cell (or unit structure) of an IRS commonly consists of electrical elements for phase modulation. However, by employing phase modulation alone, an IRS can steer the reflected electromagnetic waves toward only discrete and specific angles, leaving a wide range of out-of-beam areas. In this work, an IRS that uses both phase modulation and space modulation is presented to improve the beam resolution and continuously cover out-of-beam areas that phase modulation alone cannot address. A positive-intrinsic-negative diode is mounted on a unit cell for phase modulation, and a 4D-printed reconfigured structure is fabricated to demonstrate space modulation. The beam-steering function is achieved by alternating the states of the diodes in the same columns, while the beam resolution is improved by controlling the gaps between the columns. The functions are first theoretically and numerically analyzed and then experimentally verified, demonstrating that additional angles of −46°/+50°, −22°/+14°, and −16°/+12° are achieved with space modulation and −60°/+62°, −30°/+22°, and ±16° are achieved by phase modulation alone. The proposed IRS offers the possibility of functional integration in a variety of indoor applications within the wireless communication field.

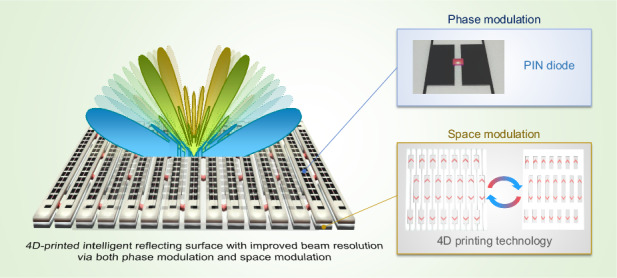

## Introduction

Recently, the need for fifth- and sixth-generation (5 G/6 G) wireless communication technologies that offer enhanced communication capabilities has emerged and developed. As these technologies operate at relatively high frequencies, maintaining the line of sight (LOS) has become a critical challenge due to signal attenuation and obstruction caused by buildings, trees, and even moisture^1^. Although radio frequency (RF) repeaters and relays have been developed to address this problem owing to their coverage competence and capacity extension, their reliance on multiple amplifiers results in high energy consumption and complex signal processing^2,3^. As a result, intelligent reflecting surfaces (IRSs) have recently been examined in many works as a possible solution to LOS issues^4–12^.

An IRS is a planar structure capable of manipulating the direction of reflected electromagnetic waves; this enables effective beam steering as well as the generation of a virtual LOS link to circumvent obstacles between the receiver and transceiver. Moreover, because IRSs do not require any transmission RF chains and are free from noise amplification and self-interference, this approach results in less energy consumption with a low profile than that of the previously mentioned solutions^2^.

The working principle of an IRS is identical to that of a reconfigurable metasurface (RMS), given that IRSs originate from RMSs. Both IRSs and RMSs employ phase modulation to reconfigure the phase of each unit cell or unit structure^13,14^ via electrical elements, such as positive-intrinsic-negative (PIN) diodes. The electrical elements offer several advantages, including dynamic tunability and beam-steering capability. Accordingly, many studies have employed one^15–17^, two^18–21^, or multiple PIN diodes per unit cell^22,23^. However, the use of PIN diodes has crucial limitations. When only one PIN diode is used, the reflected beam can be steered to only discrete and specific angles rather than a continuous range according to the theoretical equation, which is discussed in more detail in Section 2. This could be critical in terms of communication because limited reflected beam angles (due to low resolution) leave a wide out-of-beam area. Furthermore, although achieving a high-resolution phase distribution (which contributes to improved beam resolution during reflection) is possible with two or more PIN diodes, more intricate biasing circuits are needed.

To solve this problem, varactor diodes have been employed in several studies^24–31^. In another study, an RMS using a varactor diode for each unit cell in the K-band was presented^32^. A narrow beam-steering range with high resolution was achieved by applying different reverse bias voltages to control the capacitances of the varactor diode. Furthermore, a multifunctional RMS using two varactor diodes per unit cell has been proposed to continuously tune the reflected phase^33^. Nevertheless, compared with switchable PIN diodes, varactor diodes require a precise voltage biasing circuit with an additional circuit board (such as a microcontroller) to apply an exact voltage for a particular capacitance, resulting in increased complexity. Moreover, excessive power levels applied to varactor diodes can result in higher junction temperatures and performance degradation. In comparison, PIN diodes have better power-handling capabilities^34^. As a result, in this study, we aimed to find a solution to overcome the previously mentioned limitations associated with using PIN diodes.

Moreover, mechanically modified RMSs have also been researched extensively for their advantages regarding complexity, cost-effectiveness, and continuous tunability. Because RMSs deploying electrical elements require biasing circuits, which increase in complexity as the number of elements increases, mechanical methods significantly reduce this complexity. In addition, they do not require costly components, whereas electrical elements such as PIN diodes and varactor diodes necessitate expensive budgeting. Most importantly, mechanical RMSs offer continuous tunability. Since they can mechanically alter their physical geometries or shapes in a continuous manner, the electromagnetic properties can also be continuously reconfigured. In this context, structures for stretching and compressing, such as origami and kirigami, have been proposed in numerous studies^35–41^. For example, space modulation using a kirigami structure for beam-steering functionality has been previously introduced^42^. By adjusting the distance between each metastrip, the incident electromagnetic wave is reflected to different angles, which cannot be achieved without adjustment. Inspired by this work, we first propose an IRS that operates at 27.2 GHz via both phase modulation and space modulation. The limitation of discrete beam steering due to the low resolution when a single PIN diode is used is solved by adapting space modulation. One PIN diode per unit cell is employed for phase modulation, whereas a four-dimensional (4D) printed structure is selected for space modulation to demonstrate functionality due to its versatility and shape-morphing capability^43–45^. We demonstrate theoretically, numerically, and experimentally that the proposed IRS can enhance the beam resolution to continuously steer a reflected beam. This paper demonstrates the potential application of 5 G/6 G indoor wireless communication through the functional integration of two different types of modulation.

## Results

### Theory and numerical simulation

When electromagnetic waves fall vertically on an IRS in which columns are binary-coded, resulting in a phase difference of 180°, the reflected beam angle *θ*_*r*_ can be theoretically predicted as follows:

1 $${\theta }_{r}={\sin }^{-1}\left(\frac{1}{2}\cdot \frac{\lambda }{n\cdot a}\right)$$

where *n* is the number of serial unit cells with the same phase and *a* is the size of a unit cell.

**Fig. 1:**
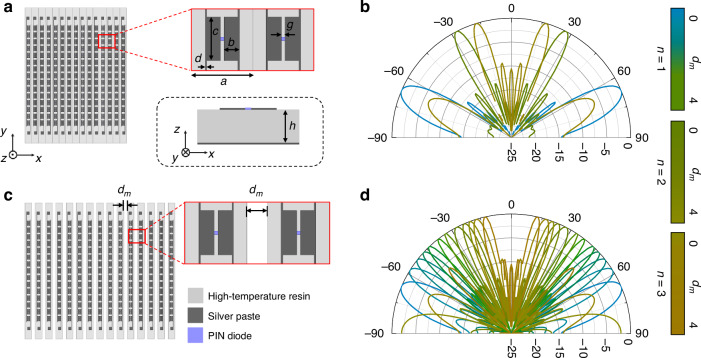
Proposed intelligent reflecting surface using both phase modulation and space modulation. **a** Intelligent reflecting surface using only phase modulation. **b** Theoretical scattering patterns using only phase modulation. **c** Intelligent reflecting surface via both phase modulation and space modulation. **d** Theoretical scattering patterns via both phase modulation and space modulation

Derived from the array factor, Eq. (1) demonstrates that phase modulation can be achieved by varying *n* and applying it to the IRS, as depicted in Fig. 1a. However, with only phase modulation, the reflected beam can be steered only to discrete angles determined by a positive integer *n*, which corresponds to the states of the PIN diodes on each column. In other words, as indicated in Fig. 1a, when the parameters in a unit cell are set to *a* **=** 6 mm, *b* **=** 1.5 mm, *c* **=** 4.4 mm, *d* **=** 0.15 mm, *g* **=** 0.36 mm, and *h* **=** 1.95 mm with an operating frequency of 27.2 GHz, *n* is the only variable in Eq. (1). For example, when the PIN diodes on one column are turned on while those on the adjacent column are turned off and this pattern is repeated periodically, *n* is determined to be 1. Similarly, when the states of PIN diodes on two columns are repeated periodically, *n* is determined to be 2. Accordingly, the beam can be steered to ±66.8°, ±27.4°, or ±17.8° when *n* is 1, 2, or 3, respectively (Fig. 1b). However, this results in a wide out-of-beam area. Hence, we propose another equation for an IRS that uses both phase modulation and space modulation, represented as

2 $${\theta }_{r}={\sin }^{-1}\left(\frac{1}{2}\cdot \frac{\lambda }{n\cdot \left(a+{d}_{m}\right)}\right)$$

where *d*_*m*_ denotes the distance between each column (namely, space modulation), as illustrated in Fig. 1c. By adjusting both *n* and *d*_*m*_, the proposed Eq. (2) reveals that employing both phase modulation and space modulation can improve the beam resolution to enhance the beam coverage in a continuous manner, which was not achievable with Eq. (1). Figure 1d displays the theoretical scattering patterns achieved with various combinations of both phase modulation and space modulation (see Fig. S1, Supplementary information).

**Fig. 2:**
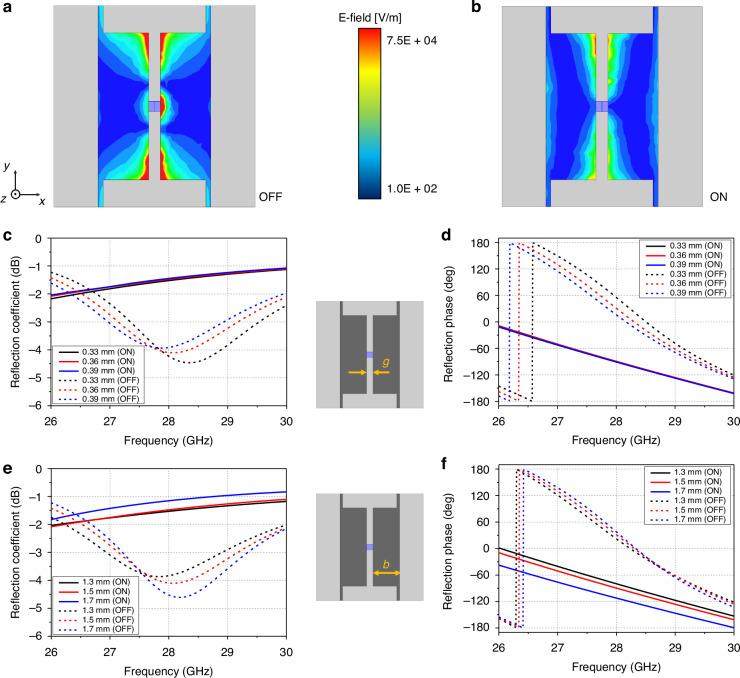
Unit cell analysis. **a** E-field distribution in the off state. **b** E-field distribution in the on state. **c** Reflection coefficient with varying *g*. **d** Reflection phase with varying *g*. **e** Reflection coefficient with varying *b*. **f** Reflection phase with varying *b*

To analyze the effects of certain geometric parameters, the electric field (E-field) distribution on the unit cell is plotted in Fig. 2a and b for both the on and off states of the PIN diode. When the diode is off, a high capacitance is produced due to the gap between the terminals (anode and cathode), whereas the capacitance is reduced when the diode is on. Hence, we analyzed the geometrical parameter *g* because the E-field is highly concentrated near the gap. As shown in Fig. 2c and d, the reflection coefficient and phase vary in the off state as we change *g*, while only a trivial variation is observed in the on state. A phase difference of 156.7° is obtained for *g* = 3.9 mm, so we choose *g* = 3.6 mm. In addition, parameter *b* is also analyzed because the direction of the E-field is identical to the direction in which *b* increases. Both the reflection coefficient and phase are changed, as shown in Fig. 2e and f, with varying *b*. Although the reflection coefficients increase for both the on and off states, the phase difference increases from 169.2° (*b* = 1.3 mm) to over 200° (*b* = 1.9 mm). Therefore, we design a unit cell with *b* = 1.6 mm.

**Fig. 3:**
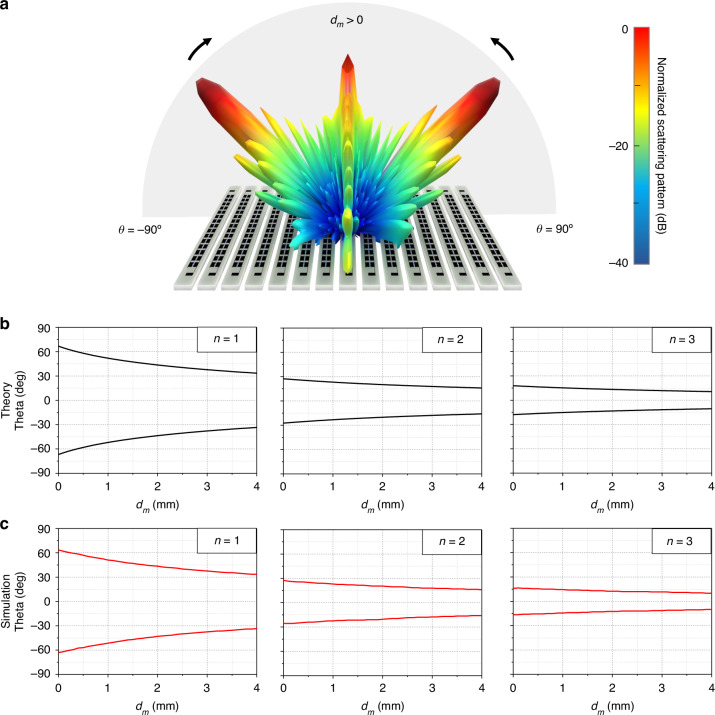
Beam-steering angles for phase modulation and space modulation. **a** Schematic indicating the variation in the beam-steering angle for different space modulations. **b** Theoretical beam-steering angles for different space modulations with phase modulations of *n* = 1, 2, and 3. **c** Simulated beam-steering angles for different space modulations with phase modulations of *n* = 1, 2, and 3

It should be noted that to ensure optimal performance, the phase difference between the on and off states of a unit cell should be maintained at close to 180°. Notably, the reflected beam angle decreases as *d*_*m*_ increases (Fig. 3a). Therefore, we simulated the unit cell to examine the reflection coefficients and phase differences when *d*_*m*_ was varied from 0 to 4 mm, which revealed that the air gaps between the columns did not significantly affect the reflection coefficients and phases, as depicted in Fig. S2 in the Supplementary Information. Figure 3b displays the theoretical beam-steering angles for different values of *d*_*m*_ when *n* = 1, 2, and 3. According to Eq. (2), the angles vary from ±66.8° at *d*_*m*_ = 0 mm, passing through ±43.6° at *d*_*m*_ = 2 mm, to ±33.5° at *d*_*m*_ = 4 mm when *n* = 1. Additionally, when *n* = 2, the angles range from ±27.4° at *d*_*m*_ = 0 mm to ±20.2° at *d*_*m*_ = 4 mm. Similarly, angles ranging from ±17.8° at *d*_*m*_ = 0 mm to ±14.5° at *d*_*m*_ = 4 mm are predicted for *n* = 3. We designed the proposed IRS consisting of 15×15 unit cells with PIN diodes and performed numerical simulations to demonstrate the improvement in the functionality of theoretical beam coverage for both phase modulation and space modulation. As a result, both the theoretical predictions and numerical simulations were identical, as displayed in Fig. 3c.

### Fabrication

**Fig. 4:**
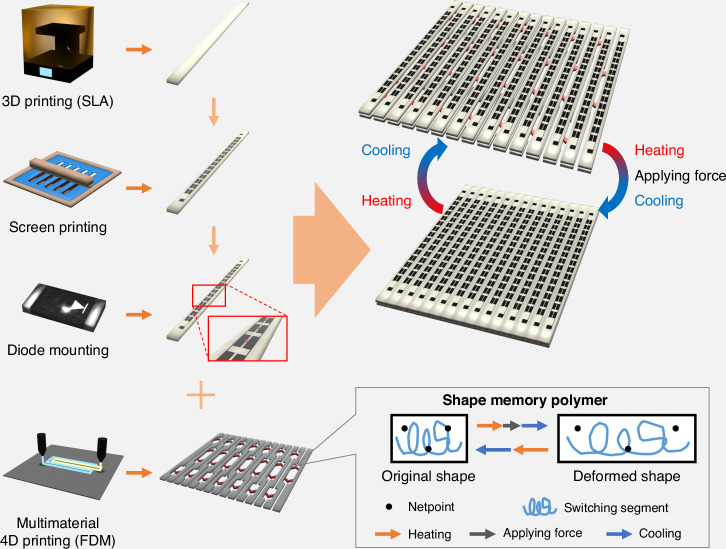
Schematic of the fabrication process, including SLA 3D printing, screen printing, diode mounting, and FDM 4D printing. The box presents the principle of a shape memory polymer, which can be deformed and restored through heating, applying force, and performing cooling processes

To validate the theoretical and numerically simulated results, the proposed IRS was fabricated. Figure 4 shows a schematic of the fabrication process (comprising several steps) and the fabricated sample. The fabrication process involved stereolithography (SLA) 3D printing, screen printing, diode mounting, and fused deposition modeling (FDM) 4D printing. Because each column was controlled for both phase modulation and space modulation, substrate columns were initially fabricated via an SLA 3D printer. We selected high-temperature durable resin with a dielectric constant of 2.8 and a loss tangent of 0.04 as the substrate material for heat endurance. Next, silver paste was screen printed on the substrates to form conductive patterns. Biasing lines and pads in the conductive patterns were considered in the fabrication process as well as in the numerical simulation to apply a voltage to each column. The ground on the bottom was also coated with silver paste, and all the conductive patterns were subsequently sintered. More details of this process are presented in the Materials and Methods section. The PIN diodes were then manually mounted on the fabricated substrate columns for phase modulation and connected to the conductive patterns using silver epoxy considering each terminal. Each column had 15 unit cells, and a total of 225 diodes were used. To perform space modulation, a reconfigured structure was also fabricated via multimaterial FDM 4D printing. The structure consisted of high-temperature and SMP filaments that were used as supports and hinges, respectively (Fig. S3, Supplementary information). Finally, the substrate columns were glued on the reconfigured structure to form the full structure. As displayed in the bottom right corner of Fig. 4, the SMP can be deformed at temperatures greater than 55 °C and can maintain its shape after cooling^46^. Furthermore, it returns to its original shape after the heating and cooling processes. Given these properties, the fabricated sample, which was initially created with a space modulation of *d*_*m*_ = 2 mm, could recover to *d*_*m*_ = 2 mm after being deformed to *d*_*m*_ = 0 mm.

### Measurement

**Fig. 5:**
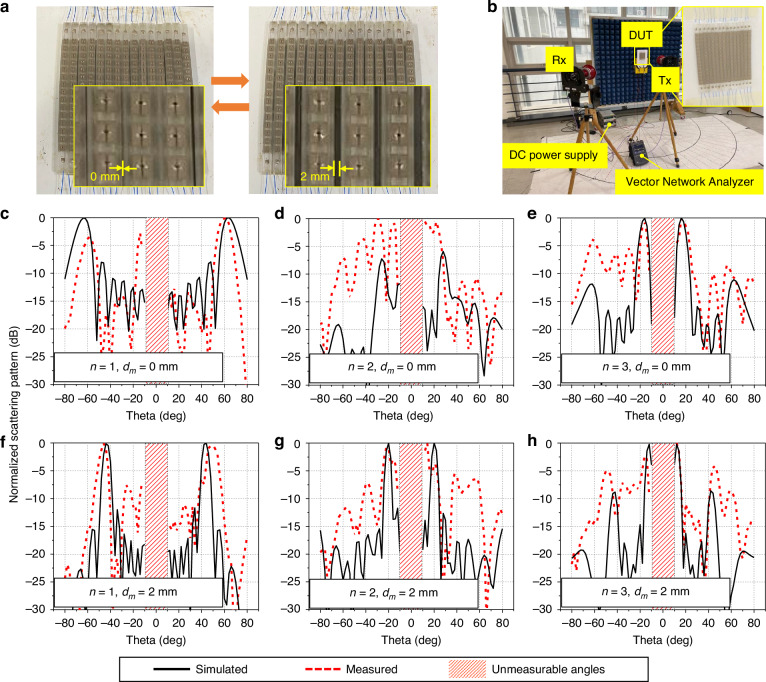
Fabricated sample and experimental validation of the intelligent reflecting surface. **a** Fabricated sample for space modulations of 0 and 2 mm. **b** Measurement setup for scattering patterns. **c–e** Normalized scattering patterns obtained using phase modulations with *n* = 1, 2, and 3 for a space modulation of 0 mm. **f–h** Normalized scattering patterns obtained via phase modulations with *n* = 1, 2, and 3 for a space modulation of 2 mm

As mentioned previously, two states of space modulation (*d*_*m*_ = 0 and 2 mm) were considered to experimentally demonstrate the proposed improved beam resolution for enhancing beam coverage. Figure 5a presents the fabricated sample with both states of space modulation. First, the sample was heated to 65 °C; then, it was deformed via an external force and cooled to room temperature to perform a space modulation of *d*_*m*_ = 0 mm. Similarly, we restored the space modulation back to *d*_*m*_ = 2 mm by heating and cooling the sample (Video S1, Supplementary Information). The electric wires were connected to the pads on the conductive patterns to apply a voltage to each column. The measurement setup used to characterize the functionality of the proposed IRS is shown in Fig. 5b. We placed a transmitting lens horn antenna (Tx) facing the sample and rotated a receiving lens horn antenna (Rx) along the protractor to measure the scattering patterns. The sample was also connected to power supplies to perform phase modulation by manipulating the states of the PIN diodes. After the experiments were completed, the results of the simulations and measurements were compared, as presented in the simulated results in Fig. S4 (Supplementary Information). Figures 4c–e and f–h present the simulated and measured results of the normalized scattering patterns for several combinations of both phase modulation and space modulation. Angles from −8° to +8° were unmeasurable because Tx and Rx overlapped and blocked the signal path, as depicted in Fig. S5 (Supplementary Information). Figures 5c and f present the results for the case of phase modulation with *n* = 1, where the space modulation *d*_*m*_ was either 0 mm or 2 mm. When the space modulation *d*_*m*_ was 0 mm, the simulated beam-steering angles were ±64°, and the measured angles were −60° and +62°. However, along with a space modulation of 2 mm, the simulated angle was ±44°, and the measured angles were −46° and +50°, enhancing beam coverage. Similarly, Fig. 5d and g display the results of phase modulation with *n* = 2 for different space modulations. The simulated and measured beam-steering angles were −26° and +28° and −30° and +22°, respectively, when *d*_*m*_ was 0 mm. When *d*_*m*_ was 2 mm, the simulated angles were ±20°, and the measured angles were −22° and +14°. Finally, the beam-steering and coverage-enhancing capabilities when *n* = 3 are shown in Fig. 5e and h. When *d*_*m*_ = 0 mm, both the simulated and measured angles were ±16°. For the case where *d*_*m*_ = 2 mm, the simulated angles were ±12°, and the measured angles were −16° and +12°. Owing to mechanical errors from the fabricated sample and measurement errors, there is a discrepancy between the experimental and simulation results at the sidelobe level. To reduce this discrepancy, precise control of *d*_*m*_ can help. In addition, a longer distance between the antenna and a larger sample (Fig. 5b) can reduce the sidelobe level and increase the main lobe level.

Although there was little difference due to the low resolution of fabrication, the measured results were in good agreement with the simulated results, confirming the promising beam coverage-enhancing functionality achieved with both phase modulation and space modulation.

## Discussion

In this article, we propose an IRS that uses both phase modulation and space modulation to improve the beam resolution for enhanced beam coverage, which is not achievable with sole reliance on phase modulation via a single PIN diode. The performance was theoretically predicted using an advanced equation derived from the conventional equation. Furthermore, we numerically showed that the beam coverage was continuous, which was attributed to the confluence of space modulation. In other words, the beam-steering capability was obtained via phase modulation using PIN diodes, and the beam resolution was improved via space modulation controlling the gaps between each column. Although the simulated results show considerable parasitic reflections in the specular (0°) direction^47^, the increase in beam resolution could still be clearly observed (Fig. S4, Supplementary Information). Therefore, we fabricated the proposed IRS using a reconfigured structure fabricated with FDM 4D printing technology to demonstrate its performance. The reconfigured structure had two states corresponding to space modulations of 0 and 2 mm. Finally, we experimentally verified that the measured results of the fabricated sample were in good agreement with the simulated results. Because the proposed IRS prototype uses 4D printing technology for demonstration, it operates slowly, requiring 240 s to recover from space modulations of 0 to 2 mm. Thus, it is difficult to deploy under dynamic conditions such as vehicle communications. However, rapid switching is not necessarily required in indoor environments. Therefore, this work offers promising potential for applications in 5 G/6 G indoor wireless communication because of its improved beam resolution, which is attributed to the combination of phase modulation and space modulation^48,49^. Moreover, practical limitations such as the high Tg of SMP materials can be addressed with the development of new SMP materials, such as a polyurethane-based SMP^50^.

## Materials and methods

### Simulations

All the numerical simulations were performed via the Ansys high-frequency structure simulator (HFSS). To simulate the periodic boundary conditions of unit cells, master/slave boundaries and Floquet ports were utilized. Moreover, perfectly matched layer (PML) boundaries and a normal incident plane wave were employed to simulate the full structure of finite 15×15 unit cells with a reconfigured structure for far-field scattering patterns. The high-temperature filaments used as supports in the reconfigured structure were set to have a dielectric constant of 2.4 and a loss tangent of 0.03, whereas the SMP filaments used as hinges were set to have a dielectric constant of 4 and a loss tangent of 0.03. We assigned a lumped RLC as an equivalent circuit to mimic the PIN diode. The PIN diode had a series inductance of 0.05 nH connected to a resistance of 5.2 Ω in the on state, whereas it had a series inductance of 0.05 nH connected to a capacitance of 0.085 pF and a resistance of 40 kΩ in parallel in the off state.

### Fabrication

A Formlabs Form3 SLA 3D printer was utilized to fabricate the substrate columns. The columns were subsequently washed with isopropyl alcohol for 20 min. Although the resin-printed structures were generally cured under ultraviolet (UV) light, the columns were not cured in this way because they tended to bend when exposed to UV light. Silver paste (Dycotec DM-SIP-2002) was screen printed on the substrate columns via a Sungjin Technologies SJ-7000M hand screen printer, after which the columns were sintered on a 50 °C hot plate. The PIN diodes (MA4GP907), which can be used as a millimeter wave-switching component, were mounted on the columns. The deviations caused by manual mounting may limit uniformity. Nevertheless, the effect is negligible because only 6 out of 225 unit cells (2.7%) deviate by more than 0.3 mm, which severely degrades the modulation quality (Fig. S6, Supplementary Information). In addition, the reconfigured structure was fabricated via a Sindoh 3DWOX 7X 3D printer that could simultaneously print two different materials. The nozzle temperatures were set to 250 and 210 °C, and the printing speeds were set to 80 and 10 mm/s for the HT and SMP filaments, respectively. The reconfigured structure was tested for long-term effects and was found to be stable (Fig. S7, Supplementary Information).

### Measurement

Ka-band lens horn antennas (LHA-30-WR28) were used to measure the scattering patterns, as depicted in Fig. 5b. In addition, a Keysight N9951A FieldFox handheld microwave analyzer was used for the measurements. Two DC power supplies were employed to perform phase modulation by turning the diodes on and off. The columns in the on state were biased at 1.37 V, while the columns in the off state were biased at 0 V. In the reconfiguring process, the prototype was placed on a 65 °C hot plate to perform space modulation. The PIN diodes performed properly because the temperature was within the operating temperature range from −55 °C to +125 °C.

## Supplementary information

Supplementary video

Supplemental Material File #1
