# Supplementary material for: 4D-printed intelligent reflecting surface with improved beam resolution via both phase modulation and space modulation
Source: Microsyst Nanoeng. 2024 Oct 29;10:157. doi: 10.1038/s41378-024-00795-1 (PMC11519457; doi:10.1038/s41378-024-00795-1)
Supplement: Supplementary file 2 — Supplemental Material File #1 [file 41378_2024_795_MOESM2_ESM.docx]

**Supplementary Information**

**4D-printed intelligent reflecting surface with improved beam resolution via both phase modulation and space modulation**

Kyounghwan Kim, Ratanak Phon, Eiyong Park, Sungjoon Lim*

Kyounghwan Kim, Sungjoon Lim

Department of Intelligent Semiconductor Engineering, Chung-Ang University, Seoul, 06974, Republic of Korea

Ratanak Phon, Eiyong Park, Sungjoon Lim

School of Electrical and Electronic Engineering, Chung-Ang University, Seoul, 06974, Republic of Korea

*Corresponding Author: Sungjoon Lim (sungjoon@cau.ac.kr)


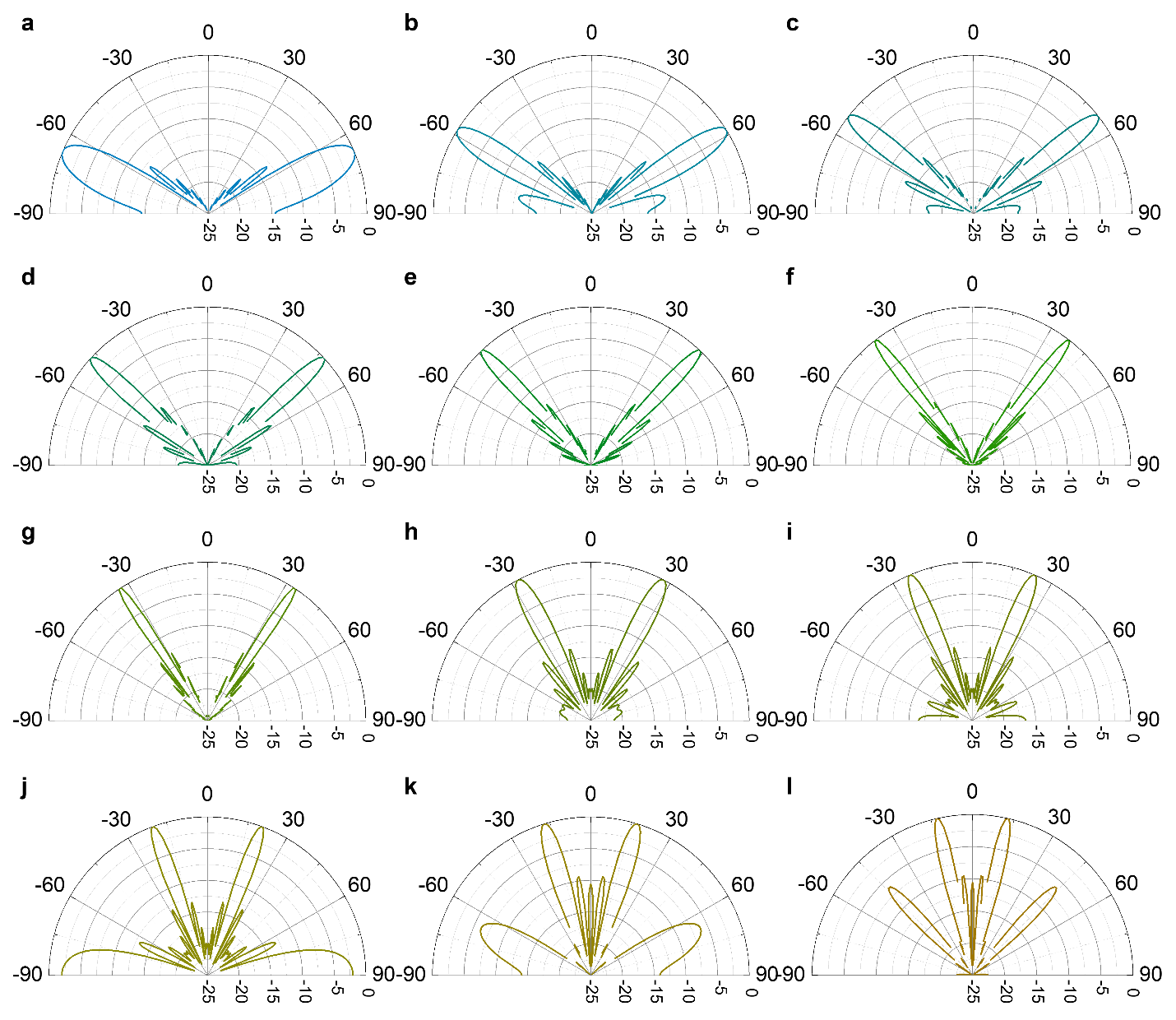


**Fig. S1** **Theoretical normalized scattering patterns obtained using both phase modulation and space modulation.** **a** Phase modulation of *n* = 1 and space modulation of $d_{m}$ = 0 mm. **b** *n* = 1 and $d_{m}$ = 0.5 mm. **c** *n* = 1 and $d_{m}$ = 1 mm. **d** *n* = 1 and $d_{m}$ = 1.5 mm. **e** *n* = 1 and $d_{m}$ = 2 mm. **f** *n* = 1 and $d_{m}$ = 3 mm. **g** *n* = 1 and $d_{m}$ = 4 mm. **h** *n* = 2 and $d_{m}$ = 0 mm. **i** *n* = 2 and $d_{m}$ = 1 mm. **j** *n* = 2 and $d_{m}$ = 2 mm. **k** *n* = 3 and $d_{m}$ = 0 mm. **l** *n* = 3 and $d_{m}$ = 2 mm.


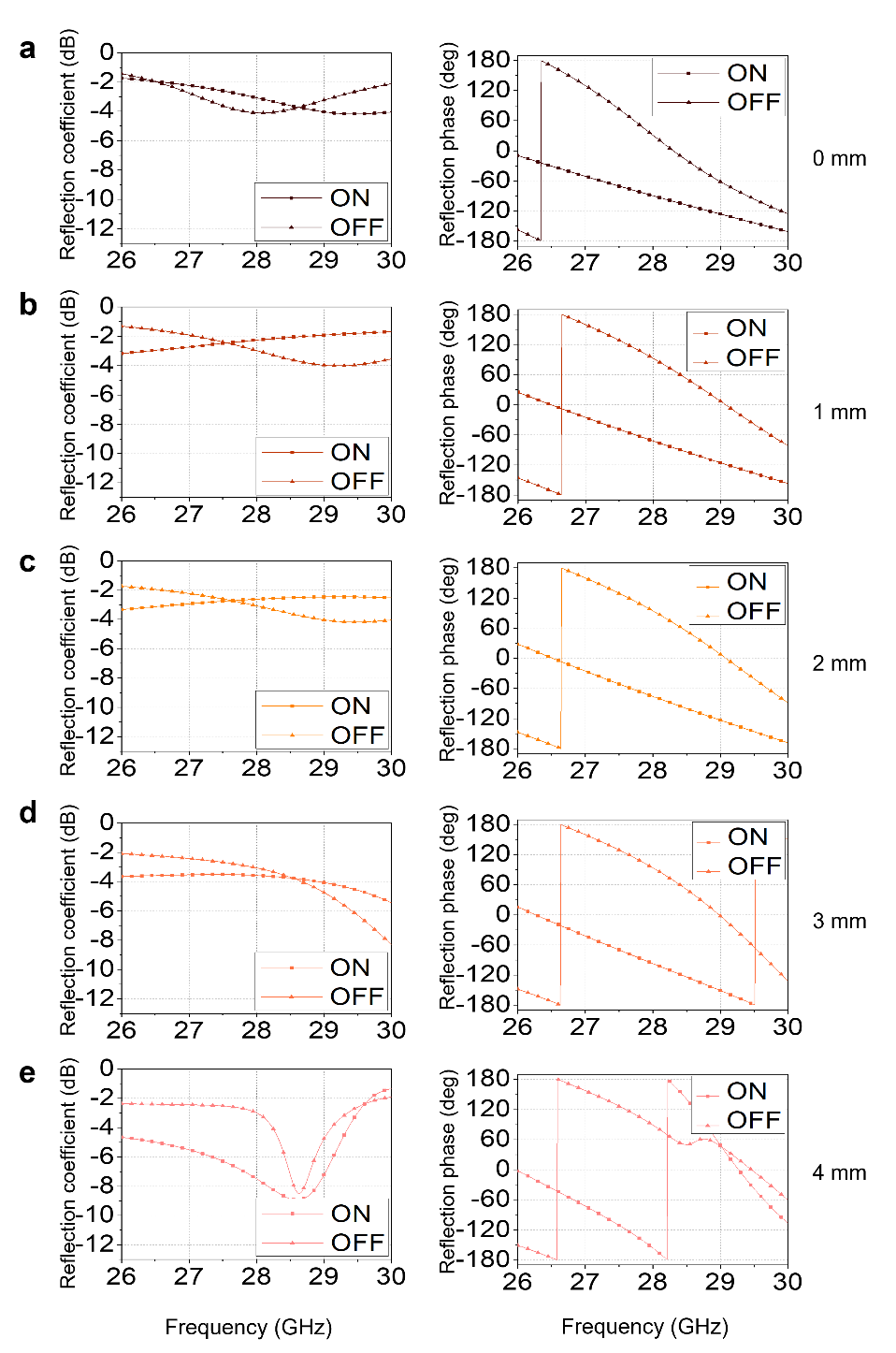


**Fig. S2** **Unit cell analysis.** **a-e** Reflection coefficient and phase in the on/off states with space modulations of 0 mm, 1 mm, 2 mm, 3 mm, and 4 mm.


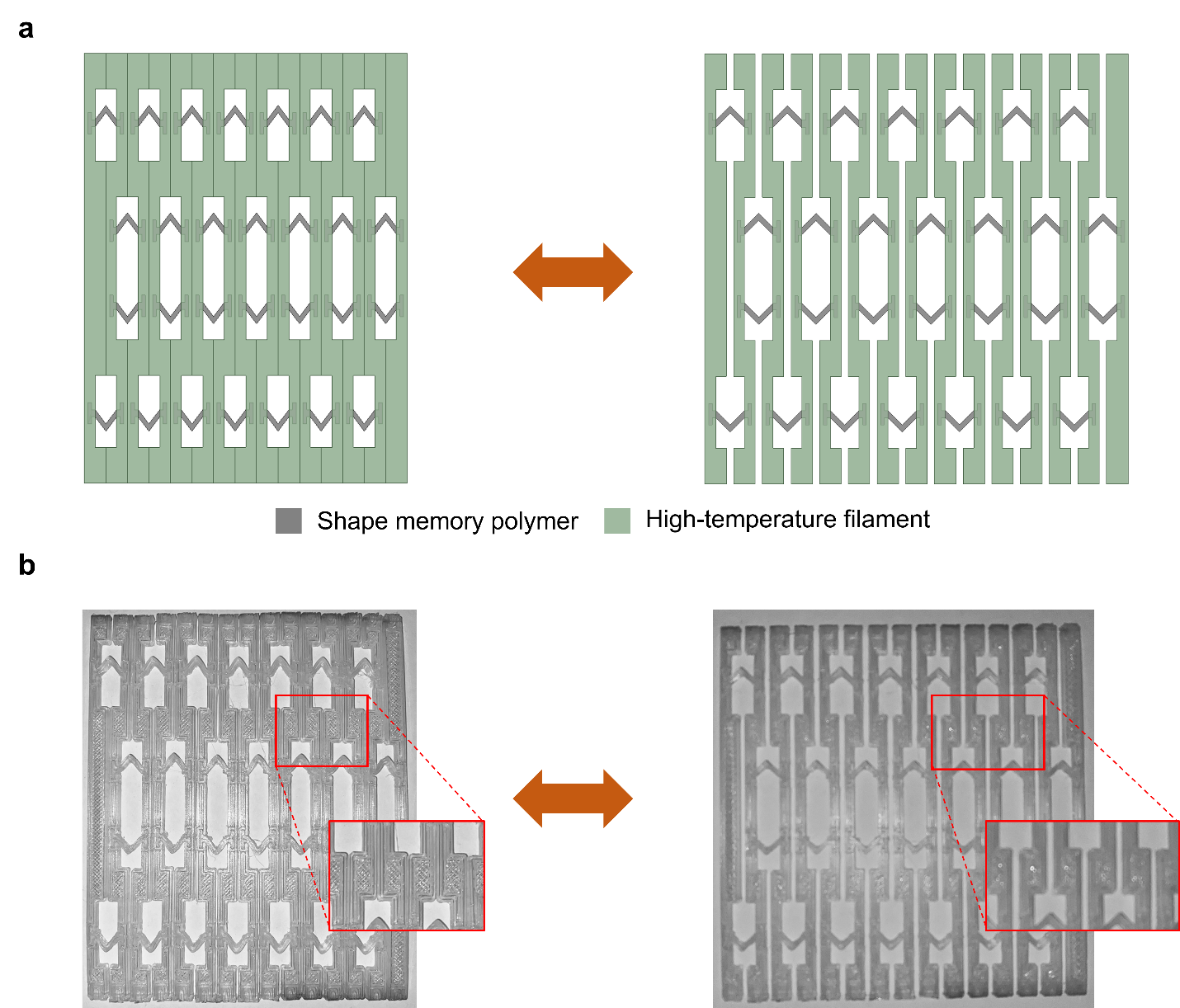


**Fig. S3** **4D-printed reconfigured structure comprising a shape memory polymer and a high-temperature filament.** **a** Schematic of the reconfigured structure. **b** Fabricated reconfigured structure.


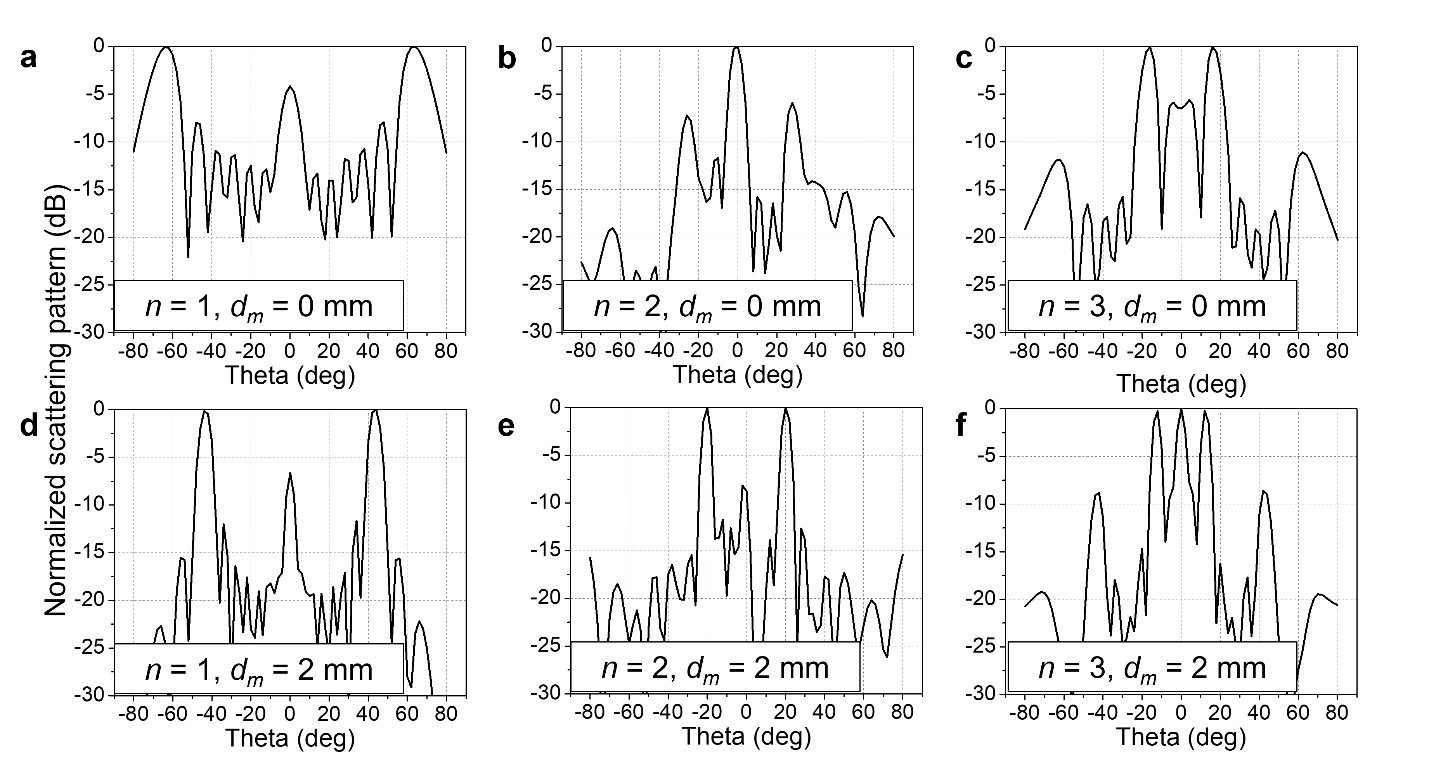


**Fig. S4** **Simulation results.** **a-c** Normalized scattering patterns obtained using a phase modulation of *n* = 1, 2, and 3 and a space modulation of $d_{m}$ = 0 mm. **d-f** Normalized scattering patterns obtained using phase modulations of *n* = 1, 2, and 3 and a space modulation of $d_{m}$ = 2 mm.


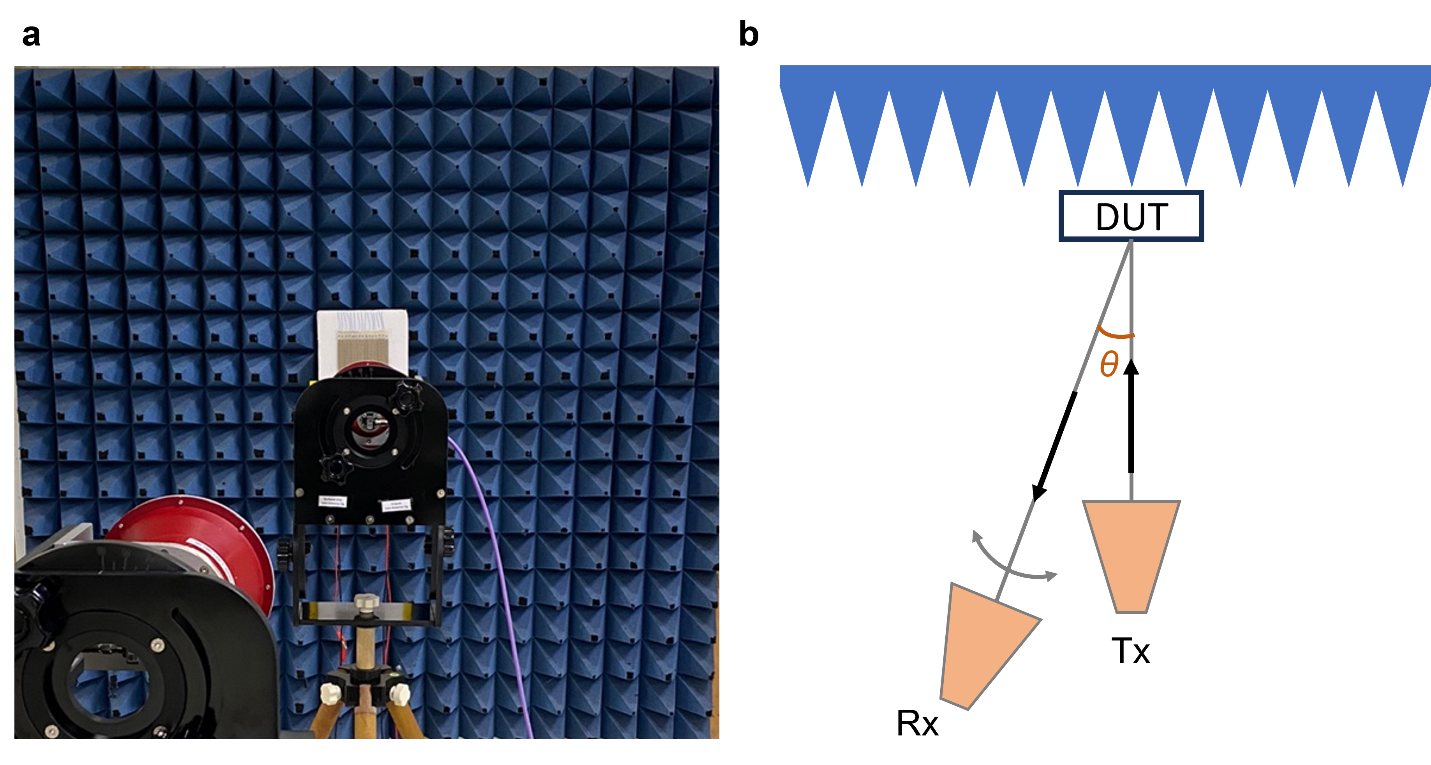


**Fig. S5** **Unmeasurable angles from** $\text{θ}$ **= -8° to 8°.** **a** Photograph of the measurement setup. **b** Illustration of the measurement setup.


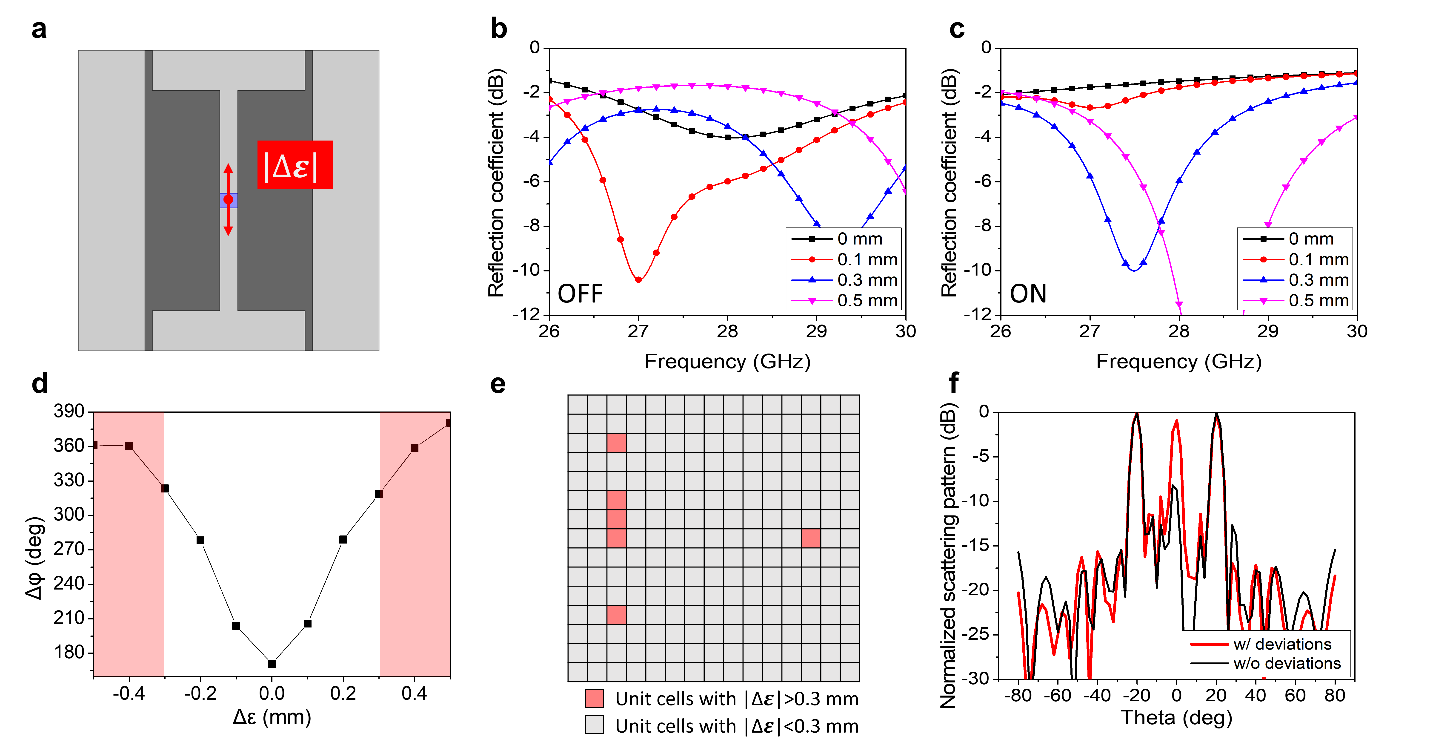


**Fig. S****6 Analysis of manually mounted PIN diodes. a** Unit cell layout that indicates the deviation of a PIN diode. **b–c** Magnitude of the reflection coefficient in the off and on states for different deviations ($\left| \Delta\varepsilon\right|$). **d** Phase difference of the reflection coefficient between the on/off states with varying deviations. **e** 15×15 unit cells, where unit cells with deviations greater than 0.3 mm are marked after checking each deviation from the fabricated sample. Six unit cells are marked. **f** Simulation results of the normalized scattering patterns with and without deviations. The deviations from **e** are considered.


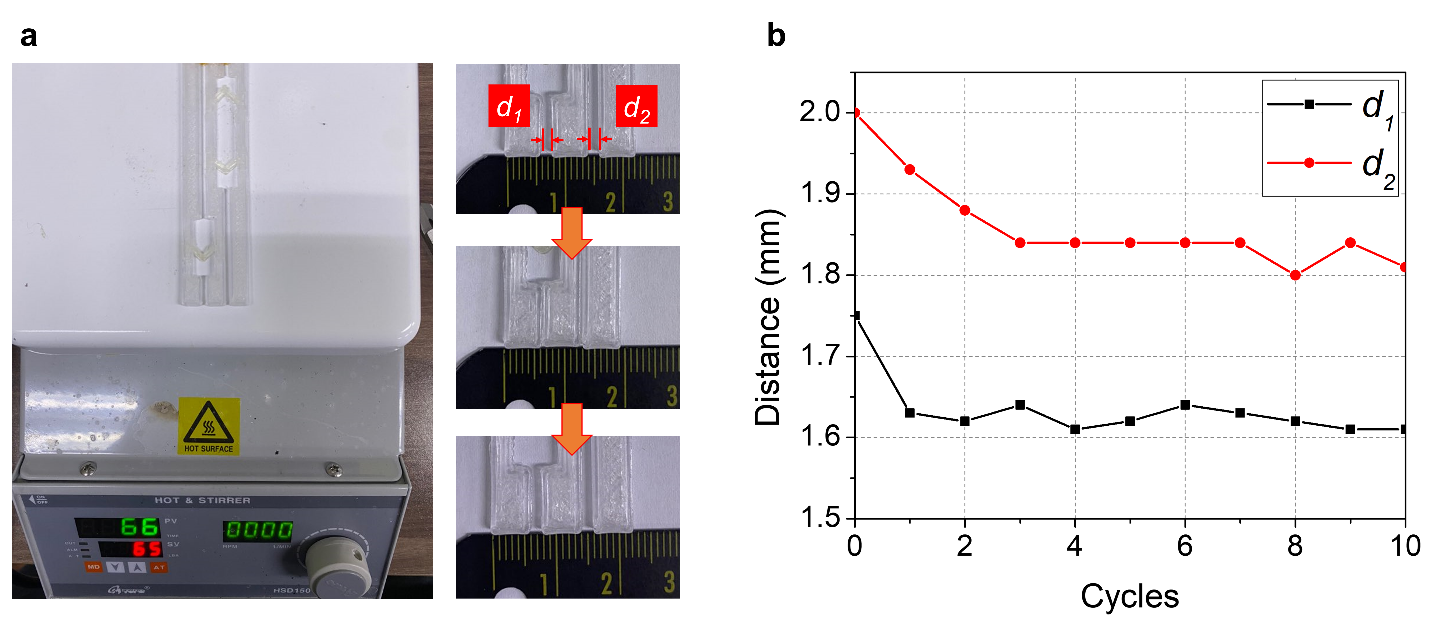


**Fig. S7 Stability test of the reconfigured structure. a** Sample with three columns and a single cycle. **b** Distance for each cycle.
